# Biomechanical Analysis of Posterior Open-Wedge Osteotomy and Glenoid Concavity Reconstruction Using an Implant-Free, J-Shaped Iliac Crest Bone Graft

**DOI:** 10.1177/03635465221128918

**Published:** 2022-10-28

**Authors:** Lukas Ernstbrunner, Paul Borbas, Andrew M. Ker, Florian B. Imhoff, Elias Bachmann, Jess G. Snedeker, Karl Wieser, Samy Bouaicha

**Affiliations:** † Department of Orthopaedic Surgery, Royal Melbourne Hospital, Parkville, Victoria, Australia; ‡ Department of Biomedical Engineering, University of Melbourne, Parkville, Victoria, Australia; § Department of Orthopaedics, Balgrist University Hospital, University of Zurich, Zurich, Switzerland; ‖ Laboratory for Orthopaedic Biomechanics, ETH Zurich, University of Zurich, Zurich, Switzerland; Investigation performed at the Balgrist University Hospital, University of Zurich, Zurich, Switzerland

**Keywords:** shoulder instability, glenoid retroversion, glenoid dysplasia, open-wedge osteotomy, posterior J-graft, biomechanics

## Abstract

**Background:**

Posterior open-wedge osteotomy and glenoid reconstruction using a J-shaped iliac crest bone graft showed promising clinical results for the treatment of posterior instability with excessive glenoid retroversion and posteroinferior glenoid deficiency.

**Purpose:**

To evaluate the biomechanical performance of the posterior J-shaped graft to restore glenoid retroversion and posteroinferior deficiency in a cadaveric shoulder instability model.

**Study Design:**

Controlled laboratory study.

**Methods:**

A posterior glenoid open-wedge osteotomy was performed in 6 fresh-frozen shoulders, allowing the glenoid retroversion to be set at 0°, 10°, and 20°. At each of these 3 preset angles of glenoid retroversion, the following conditions were simulated: (1) intact joint, (2) posterior Bankart lesion, (3) 20% posteroinferior glenoid deficiency, and (4) posterior J-shaped graft (at 0° of retroversion). With the humerus in the Jerk position (60° of glenohumeral anteflexion, 60° of internal rotation), stability was evaluated by measuring posterior humeral head (HH) translation (in mm) and peak translational force (in N) to translate the HH over 25% of the glenoid width. Glenohumeral contact patterns were measured using pressure-sensitive sensors. Fixation of the posterior J-graft was analyzed by recording graft micromovements during 3000 cycles of 5-mm anteroposterior HH translations.

**Results:**

Reconstructing the glenoid with a posterior J-graft to 0° of retroversion significantly increased stability compared with a posterior Bankart lesion and posteroinferior glenoid deficiency in all 3 preset degrees of retroversion (*P* < .05). There was no significant difference in joint stability comparing the posterior J-graft with an intact joint at 0° of retroversion. The posterior J-graft restored mean contact area and contact pressure comparable with that of the intact condition with 0° of retroversion (222 vs 223 mm^2^, *P* = .980; and 0.450 vs 0.550 MPa, *P* = .203). The mean total graft displacement after 3000 cycles of loading was 43 ± 84 µm, and the mean maximal mediolateral graft bending was 508 ± 488 µm.

**Conclusion:**

Biomechanical analysis of the posterior J-graft demonstrated reliable restoration of initial glenohumeral joint stability, normalization of contact patterns comparable with that of an intact shoulder joint with neutral retroversion, and secure initial graft fixation in the cadaveric model.

**Clinical Relevance:**

This study confirms that the posterior J-graft can restore stability and glenohumeral loading conditions comparable with those of an intact shoulder.

Operative management of posterior shoulder instability with excessive glenoid retroversion and posteroinferior glenoid deficiency is challenging and includes soft tissue procedures, posterior bone block, or glenoid osteotomy.^4,7,16,17,35^ There is general agreement that soft tissue procedures such as arthroscopic and open posterior labral repair may not be as durable in the setting of severe glenoid retroversion and posteroinferior glenoid dysplasia. Although posterior bone-block procedures can increase the width of the posteroinferior glenoid joint surface,^41^ excessive glenoid retroversion remains unchanged. This alteration in articular alignment is a known risk for posterior instability as well as persistent instability after posterior shoulder stabilization procedures.^4,5,20,22,25,27^ Correcting glenoid retroversion therefore has theoretical advantages in restoring joint stability, and using an opening-wedge osteotomy to do so was first described by Scott.^35^ Although this procedure has evolved since the original description, recent long-term results of this procedure revealed that shoulder stability was not reliably restored, and the procedure was not able to recenter the joint or prevent progression of osteoarthritis.^39^

A recent novel surgical technique was introduced that combines a posterior opening-wedge osteotomy with glenoid concavity reconstruction using an implant-free, J-shaped autograft iliac crest bone graft.^9^ The preliminary short-term results of a small group of patients with atraumatic posterior shoulder instability associated with excessive glenoid retroversion and posteroinferior glenoid deficiency showed that the posterior J-graft is able to reconstruct posteroinferior glenoid morphology, correct glenoid retroversion, and improve posterior shoulder instability.^12^ However, no biomechanical data on this technique are available.

The purpose of this study was to evaluate the (1) stabilizing effect, (2) glenohumeral contact patterns, and (3) initial graft fixation under cyclic loading, using the posterior J-graft to restore glenoid retroversion and posteroinferior deficiency in a cadaveric shoulder model. It was hypothesized that the posterior J-graft would restore stability, which was defined as the amount of posterior displacement when a posteriorly directed force of 20 N was applied as well as the force that was needed to resist posterior translation over 25% of the glenoid width, and glenohumeral contact patterns, which were assessed by using pressure-sensitive pads to measure contact area and contact pressure throughout posterior humeral head (HH) translation, to that of a native glenohumeral joint with secure initial graft fixation.

## Methods

Ethical approval was obtained for this controlled laboratory study.

### Specimen Preparation and Setup

Six fresh-frozen cadaveric shoulders and hemipelvises for iliac crest bone graft harvest from MedCure were used for this study. The mean age at death was 52.8 ± 9.2 years, and there were 3 female and 3 male donors. All specimens were inspected visually and by computed tomography (CT) scan for soft tissue or bone defects, or signs of moderate to advanced glenohumeral arthritis, and deemed to have no signs of glenohumeral arthritis or macroscopic rotator cuff tears. Further CT scan evaluation included native glenoid retroversion and bone mineral density assessment in a 1 × 1–cm area of the posterior glenoid neck.^10^ All specimens were thawed for 24 hours at room temperature. All the soft tissues were dissected off the scapula and humerus, leaving only the capsule and labrum intact. The anterior capsule was vented as described previously.^23^ The humeral shafts were cut 2 cm distal to the deltoid tuberosity. The scapulae were potted with polymethylmethacrylate in a custom box with the glenoid surface parallel to the floor (ie, 0° of glenoid retroversion). The humeri were fixed with multiple screws into a customized cylinder. Specimens were kept moist with phosphate-buffered saline to prevent dehydration during specimen preparation, surgical repair, and testing.

### Creation of Retroversion Angles and Soft Tissue and Bone Defects

**Figure 1.**
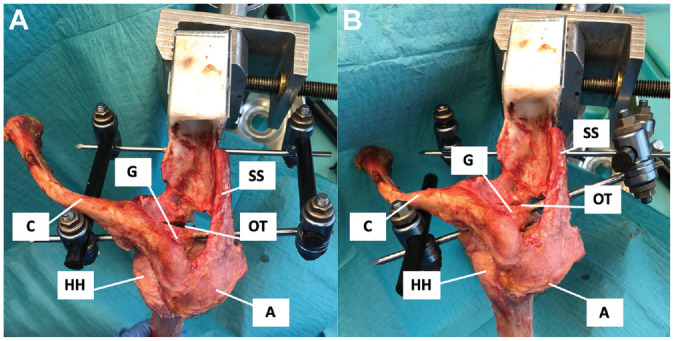
Photograph from superior showing the mounted external fixator for controlling the different angles of glenoid retroversion of a left shoulder. (A) The posterior osteotomy is opened so that glenoid retroversion is set at 0° according to the individual native version. (B) The posterior osteotomy is closed so that glenoid retroversion is set at 20°. A, acromion; C, clavicle; G, glenoid; HH, humeral head; OT, osteotomy; SS, scapular spine.

A posteroanterior 4.0-mm Steinman pin was drilled parallel to the glenoid surface into the superior third of the superoinferior diameter of the glenoid. A second 4.0-mm Steinman pin was placed medially through the scapular body, and an external fixator was mounted between the 2 pins. Next, a 20° wedge was cut from the posteroinferior glenoid neck with an oscillating saw 15 mm medial to the glenoid surface. The osteotomy was aimed into the base of the coracoid, and the anterior cortex was kept intact. Based on previous studies on retroversion as a risk factor for posterior instability,^9,19,22,23^ 3 different angles were obtained through the external fixator: 0°, 10°, and 20° of glenoid retroversion (Figure 1).

The aimed angle of retroversion was confirmed before and after each test with a 3-dimensional digitizer (MicroScribe; Immersion) with a range of ±0.5°.^23^ Each of the 6 specimens underwent the following testing conditions in chronological order:

1. Intact joint, neutral retroversion
2. Intact joint, 10° of retroversion
3. Intact joint, 20° of retroversion
4. Posterior Bankart lesion, neutral retroversion
5. Posterior Bankart lesion, 10° of retroversion
6. Posterior Bankart lesion, 20° of retroversion
7. 20% posteroinferior glenoid deficiency, neutral retroversion
8. 20% posteroinferior glenoid deficiency, 10° of retroversion
9. 20% posteroinferior glenoid deficiency, 20° of retroversion
10. Posterior J-graft creating neutral retroversion

The posterior Bankart lesion was created through the anterior capsular window. The labrum was sharply detached from 6- to 9-o’clock. Next, a longitudinal posterior capsulotomy was performed and the posteroinferior glenoid rim exposed. An osteotomy simulating 20% posteroinferior glenoid deficiency was chosen based on the preoperative glenoid morphology of patients undergoing the posterior J-graft.^12^ The exact dimension of the osteotomy was calculated using CT scan analysis based on the Pico method.^11,13,14,29^ The MicroScribe digitizer was used to mark the osteotomy, which was then performed with a chisel along a line parallel to the long axis of the glenoid.^42^

### Graft Preparation and Glenoid Reconstruction

**Figure 2.**
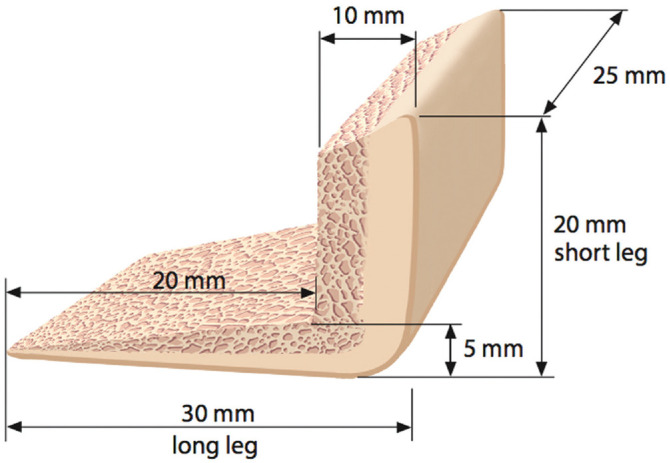
The J-shaped iliac crest bone graft typically measures 25 mm wide (superoinferior dimension of the glenoid in the en face view), 30 mm long (long leg; anteroposterior [AP] dimension), and 10 mm high (graft surface; AP dimension). The graft surface is molded to a ramplike structure to reconstruct the concavity of the posteroinferior glenoid. The dimension of the short leg of the J-graft usually measures 20 to 25 mm (mediolateral dimension). The long leg and short leg are composed of cortical bone on the outside and cancellous bone on the inside to facilitate bony ingrowth.

Two fellowship-trained shoulder surgeons (L.E., P.B.) performed all surgeries. Harvest, preparation, and implantation of the J-shaped iliac crest bone grafts were conducted according to the original surgical technique^12^: a bicortical iliac crest bone graft including the crest and inner table was harvested and molded into a J-shaped graft using the oscillating saw. The J-graft was used to correct to 0° of retroversion, and the mean width at the widest point of the long leg of the J-graft was 4.7 ± 0.7 mm (Figure 2).

**Figure 3.**
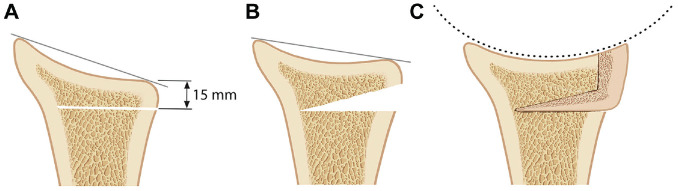
Illustration of the posterior J-graft for correction of glenoid retroversion and reconstruction of posteroinferior glenoid deficiency. (A) The osteotomy was performed 15 mm medial to the glenoid surface from posterior to anterior, aiming into the base of the coracoid with the anterior cortex kept intact. (B) The osteotomy was then opened to fit the graft. (C) The J-graft was impacted into the osteotomy site with a press fit. The ramp-shaped graft surface reconstructed the glenoid concavity and compensated the posteroinferior glenoid deficiency.

The J-graft was inserted into the osteotomy site and impacted into place with a press fit (Figure 3). The posteroinferior capsulolabral complex was repaired transosseously with No. 1 Vicryl (Ethicon, Johnson & Johnson Medtech) sutures, leaving an extra-articular graft.

### Stability Testing

**Figure 4.**
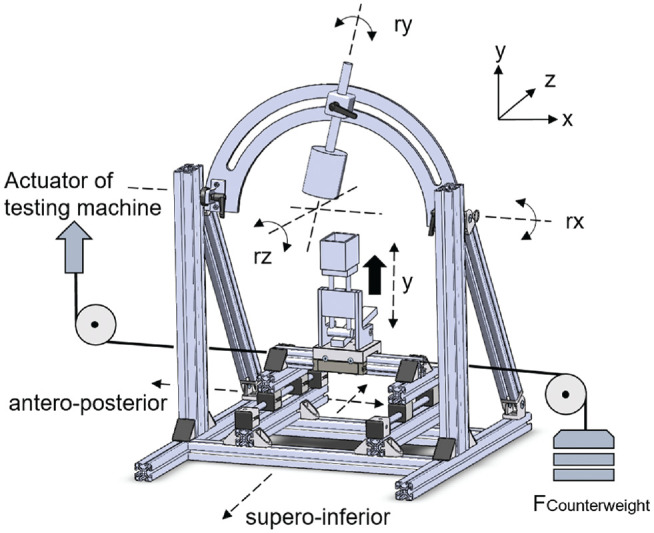
Illustration of the custom shoulder-testing system with 6 degrees of freedom of glenohumeral joint positioning. Solid arrow in black indicates joint compression force (22 N and 50 N, respectively) generated by a static weight via a lever arm. Rotation (*ry*), abduction (*rx*), and horizontal abduction (*rz*) could be set according to the testing protocol. Anteroposterior translation was generated by a universal materials testing machine (not displayed), which was connected by a steel cable running from the translation plate to the actuator of the machine. A counterweight, which was positioned opposite to said translation plate, allowed cyclic loading (respectively, automatic repositioning) when load from the machine was removed.

The specimens were mounted onto a custom shoulder-testing system with 6 degrees of freedom of glenohumeral joint positioning.^23,33,42^ The humerus was mounted in the Jerk test position,^28^ which was defined as neutral adduction, 60° of glenohumeral anteflexion, and 60° of internal rotation.^40^ The scapular box was mounted onto a vertical linear bearing translator and lever arm system on top of the 2 translation plates that permitted anteroposterior and superoinferior translations (Figure 4).

A compressive load of 22 N was applied perpendicular to the glenoid through the vertical linear bearing translator and lever arm system, allowing the HH to find its neutral position in the glenoid concavity.^23,36^ This position was defined as the reference-neutral position^33^ and recorded with a universal materials testing machine (MTS; Zwick 010; Zwick/Roell). All specimens were preconditioned for 10 cycles with a cyclic force of −10 to 10 N relative to the applied baseline compressive force.^23^ The actual stability testing was composed of 2 different protocols: (A) a force-controlled protocol analyzing the amount of posterior HH translation (in mm) with a posteriorly directed force of 20 N at a rate of 1 N/s and (B) a displacement-controlled protocol analyzing the peak translational force needed to translate the HH posteriorly over 25% of the glenoid width at a rate of 3.0 mm/s. The amplitude of force applied in protocol A was chosen according to a similar biomechanical study.^23^ The amplitude of displacement applied in protocol B was chosen according to multiple biomechanical shoulder instability studies.^26,33,42,43^ Forces observed during testing were recorded by a load cell (Xforce P; Zwick/Roell) of the MTS. All tests were performed with the humerus in the Jerk test position and at each of the 3 glenoid retroversion angles (0°, 10°, and 20°) for the following glenoid conditions: (1) intact joint, (2) posterior Bankart lesion, and (3) 20% posteroinferior glenoid deficiency. Then all tests were performed after posterior J-graft positioning correcting to 0° of glenoid retroversion. Testing was repeated 3 times, and mean values were recorded.

### Glenohumeral Contact Area and Pressure

The same shoulder-testing system as outlined above was used for measuring glenohumeral contact patterns.^23,33,42^ The 0.1 mm–thick dynamic pressure-sensitive pads (Sensor No. 4205; Tekscan) were used.^26,42^ All sensors were precalibrated according to the manufacturer’s guidelines. The sensor was positioned between the HH and glenoid concavity and marked to ensure correct positioning throughout testing.^18,33^

After finding the neutral position of the HH in the glenoid concavity as described above, the same force-controlled protocol with a posteriorly directed force of 20 N was used for analyzing glenohumeral contact patterns. The pressure-sensitive pads measured contact area and contact pressure throughout translation. Again, all tests were performed with the humerus in the Jerk test position and at each of the 3 glenoid retroversion angles (0°, 10°, and 20°) for the intact, pathological, and repaired (at 0°) conditions as outlined previously.

### J-Graft Fixation Under Cyclic Loading

Cyclic loading was also conducted with the same custom shoulder-testing system.^23,33,42^ As described in a similar biomechanical study on cyclic graft loading, the compressive force was increased to 50 N.^33^ Again, the compressive load allowed the HH to find its neutral position within the glenoid concavity, which defined the reference-neutral position.^33^ With the humerus in the Jerk test position, a 5-mm posteroanterior translation cyclic loading protocol at a rate of 4.0 mm/s was selected according to a similar study.^33^ The reconstructed glenoid and posterior J-graft were loaded for a total of 3000 cycles. During cyclic loading, graft displacement in the posterior direction (along the long leg of the J-graft) and mediolateral bending motions were recorded using 2 high-resolution (±1.5 µm precision), linear differential variable reluctance transducer strain gauges (MG-LVDT-3; LORD Sensing MicroStrain). These 2 strain gauges were placed in line with the long leg of the posterior J-graft and in the mediolateral direction, respectively.^33^

### Statistical Analysis

Distribution of the data was assessed with the Shapiro-Wilk test. All conditions in each of the 3 different angles of glenoid retroversion for stability testing and assessment of glenohumeral contact pattern were compared using the repeated-measures analysis of variance (ANOVA; parametric data) and Friedman ANOVA (nonparametric data). Significance was set as *P* < .05 with use of Bonferroni (repeated-measures ANOVA) and Dunn-Bonferroni (Friedman ANOVA) adjustments; all *P* values were 2-tailed.

An a priori power analysis revealed that for a significance level of .05 (type I error), a sample size of 6 specimens was sufficient to provide a desired power of 80% to detect a difference of >5 mm of posterior HH translation with a similar force-controlled testing protocol.^23^

## Results

Mean native glenoid retroversion was 2.6° (range, +2° to 8°), and bone mineral density was 162 ± 31 Hounsfield units.

### Stability Testing

**Figure 5.**
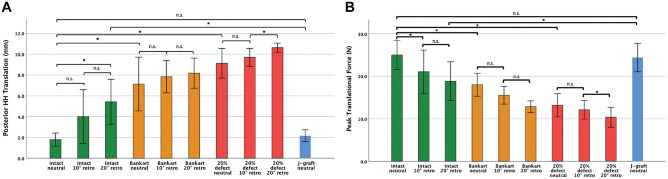
(A) The amount of posterior humeral head (HH) translation (in mm) with a posteriorly directed force of 20 N against a compressive load of 22 N with the arm in the Jerk test position is shown for the 3 glenoid retroversion (retro) angles (0°, 10°, and 20°) and the following glenoid conditions: (1) intact, (2) posterior Bankart lesion, (3) 20% posteroinferior glenoid deficiency, and (4) posterior J-graft at 0° of glenoid retroversion. (B) The peak translational force (in N) needed to translate the HH posteriorly over 25% of the glenoid width against a compressive load of 22 N with the arm in the Jerk test position is shown for the 3 glenoid retroversion angles (0°, 10°, and 20°) and the following glenoid conditions: (1) intact joint, (2) posterior Bankart lesion, (3) 20% posteroinferior glenoid deficiency, and (4) posterior J-graft at 0° of glenoid retroversion. Values are presented as mean and SE bars. *Level of significance: *P* < .005. n.s., nonsignificant.

Under both testing protocols, stability significantly decreased with increasing glenoid retroversion and with advanced posteroinferior glenoid defect size. The posterior J-graft restored stability to values comparable with the intact condition, with 0° of retroversion (posterior HH translation: 2.2 vs 1.8 mm, *P* = .339; peak translational force: 24 vs 25 N, *P* = .597). The posterior J-graft showed significantly less posterior HH translation compared with the intact condition with 20° of retroversion (2.2 vs 5.4 mm; *P* = .024) (Figure 5).

Detailed information of the results revealed during the 2 instability testing protocols can be found in the Appendix file (available in the online version of the article).

### Contact Area and Pressure

**Figure 6.**
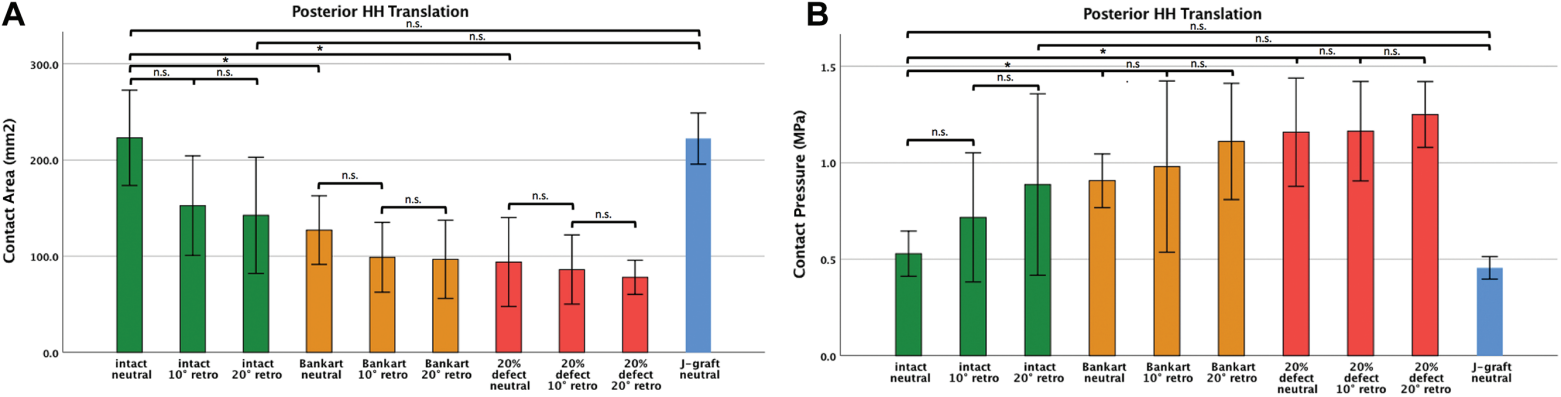
Glenohumeral (A) contact area and (B) contact pressure with the arm in the Jerk test position is shown for the 3 glenoid retroversion (retro) angles (0°, 10°, and 20°) and the following glenoid conditions: (1) intact joint, (2) posterior Bankart lesion, (3) 20% posteroinferior glenoid deficiency, and (4) posterior J-graft at 0° of glenoid retroversion. Values are presented as means and SE bars. *Level of significance: *P* < .005. n.s., nonsignificant. HH, humeral head.

The mean contact area significantly decreased with a posterior Bankart lesion and posteroinferior glenoid deficiency, respectively (Figure 6A). The posterior J-graft restored the mean contact area comparable with the intact condition, with 0° of retroversion (222 vs 223 mm^2^; *P* = .980).

Similarly, mean contact pressure significantly increased with a posterior Bankart lesion and posteroinferior glenoid deficiency, respectively (Figure 6B). The posterior J-graft also restored the contact pressure comparable with the intact condition with 0° of retroversion (0.450 vs 0.550 MPa; *P* = .203).

### J-Graft Fixation

The mean total graft displacement in the posterior direction (along the long leg of the J-graft) after 3000 cycles of posteroanterior loading was 43 ± 84 µm. The mean maximal mediolateral bending relative to the unloaded condition of the posterior J-graft during 3000 cycles of posteroanterior loading was 508 ± 488 µm.

## Discussion

The main findings of this biomechanical cadaveric study were that posterior opening-wedge osteotomy and glenoid reconstruction using an implant-free J-shaped iliac crest bone graft restored stability and normalized glenohumeral contact patterns comparable with an intact glenohumeral joint. It also demonstrated secure initial implant-free graft fixation.

Atraumatic posterior shoulder instability associated with excessive glenoid retroversion and posteroinferior glenoid deficiency remains a challenge in shoulder surgery. Neither posterior labral repair nor conventional bone-block procedures can correct the glenoid angular structural deformity. The short-term results of an original technique to correct glenoid retroversion using an opening-wedge glenoid osteotomy as described by Scott^35^ were somewhat promising.^24,32^ However, recently published long-term studies have reported that this technique fails to durably restore stability, correct posterior HH subluxation, and prevent progression of osteoarthritis.^39^

While the aforementioned techniques may not be the key to this unsolved problem, the short-term clinical and radiographic results of the novel posterior J-graft technique appear promising.^12^ This procedure combines the traditional Scott osteotomy with reconstruction of the posteroinferior glenoid concavity using an implant-free J-shaped iliac crest bone graft. The use of a J-graft is an established procedure for patients with recurrent anterior instability.^1,13^ Its main biomechanical advantage compared with other bone-block procedures is that it re-creates glenoid concavity, which not only has a stabilizing effect but also improves glenohumeral contact patterns.^15,18,31^ In comparison with conventional posterior bone-block procedures,^4^ the posterior J-graft can also treat excessive glenoid retroversion, as proven recently.^12^

The stabilizing effect of the posterior J-graft was confirmed in this biomechanical study. It not only restored stability to values comparable with those of intact condition with 0° of retroversion but also showed significantly less posterior HH translation compared with the intact condition with 20° of retroversion. Therefore, correcting excessive glenoid retroversion with the posterior J-graft appears to have an additional positive influence on stability in the setting of excessive glenoid retroversion without significant glenoid deficiency. One disadvantage related to this technique remains the donor-site morbidity, which mostly involves postoperative pain and diminished sensation around the scar.^2,3^ It would be interesting to study other bone graft sources such as distal tibial allograft^34^ or local autograft from the acromion or scapular spine.^30^

Restoration of glenohumeral congruency can normalize glenohumeral contact pressure.^18^ The current results showed that contact area significantly decreased and contact pressure significantly increased with creation of a Bankart lesion and posteroinferior glenoid deficiency. These differences were even more pronounced with increasing glenoid retroversion of 10° and 20°, respectively. On the other hand, the posterior J-graft restored native contact patterns by reconstruction of the posteroinferior glenoid concavity and correcting glenoid retroversion to 0°. The testing protocol used in this study for evaluation of glenohumeral contact patterns is unique: it considers the physiologic range of posterior HH translation^21,36^ and represents eccentric loading of the posterior glenoid, a classic finding in patients with posterior instability associated with excessive glenoid retroversion and dysplasia.^8,37,38^ Whether the posterior J-graft has the potential to slow the development or progression of osteoarthritis remains unclear, but based on this biomechanical study, it appears that it does not lead to increased contact pressure, a concern that was raised with the Scott osteotomy.^39^

The posteroinferior capsulolabral complex was repaired in this study. Yet, the role of extra-articular graft placement in glenohumeral contact patterns needs to be further investigated. With the available results from this study assessing the posterior J-graft procedure, it also remains unclear whether secondary shoulder arthroplasty is easier to perform or more successful because the glenoid version and morphology have been corrected (without the use of any metal implants).

The posterior J-graft is technically challenging. Although in the original description, no graft-related complications were observed, it must be noted that the treating surgeons were very experienced.^12^ The surgical procedures performed in this biomechanical study were conducted by 2 fellowship-trained shoulder surgeons, both trained by the senior surgeon of the original description. Cyclic loading testing of the graft in this study demonstrated that, when conducted in a technically correct manner, the posterior J-graft achieved excellent initial fixation without any graft breakage reported.

There are certain limitations associated with this biomechanical study. Static and dynamic stabilizing factors cannot be fully replicated in a cadaveric study. Furthermore, the stabilizing effect of periscapular muscles was not considered, although the capsular complex was kept intact during testing of the intact condition and after repair. The stabilizing effect of the posterior J-graft and its effect on glenohumeral contact patterns represents mainly the early postoperative condition, as the graft usually undergoes a remodeling process.^6^ Bone and soft tissue quality of the specimens used may differ from in vivo conditions, and the number of different testing conditions and testing cycles applied per specimen may have had a cumulative effect on tissue quality. Last, the magnitude of the minimum detectable difference (ie, >5 mm posterior HH translation) and the number of comparisons made are limitations of the sample size used in this study.

## Conclusion

Biomechanical analysis of the posterior opening-wedge osteotomy and glenoid reconstruction technique using an implant-free J-shaped iliac crest bone graft conducted in this study demonstrated that the procedure reliably restores stability and normalizes glenohumeral contact patterns comparable with an intact cadaveric shoulder joint with neutral retroversion. Furthermore, secure initial implant-free graft fixation was found in the cadaveric model.

## Supplemental Material

sj-pdf-1-ajs-10.1177_03635465221128918 – Supplemental material for Biomechanical Analysis of Posterior Open-Wedge Osteotomy and Glenoid Concavity Reconstruction Using an Implant-Free, J-Shaped Iliac Crest Bone Graft Click here for additional data file. Supplemental material, sj-pdf-1-ajs-10.1177_03635465221128918 for Biomechanical Analysis of Posterior Open-Wedge Osteotomy and Glenoid Concavity Reconstruction Using an Implant-Free, J-Shaped Iliac Crest Bone Graft by Lukas Ernstbrunner, Paul Borbas, Andrew M. Ker, Florian B. Imhoff, Elias Bachmann, Jess G. Snedeker, Karl Wieser and Samy Bouaicha in The American Journal of Sports Medicine
