# Supplementary material for: Biomechanical Analysis of Posterior Open-Wedge Osteotomy and Glenoid Concavity Reconstruction Using an Implant-Free, J-Shaped Iliac Crest Bone Graft
Source: Am J Sports Med. 2022 Oct 28;50(14):3889–96. doi: 10.1177/03635465221128918 (PMC9729978; doi:10.1177/03635465221128918)
Supplement: sj-pdf-1-ajs-10.1177_03635465221128918 – Supplemental material for Biomechanical Analysis of Posterior Open-Wedge Osteotomy and Glenoid Concavity Reconstruction Using an Implant-Free, J-Shaped Iliac Crest Bone Graft [file sj-pdf-1-ajs-10.1177_03635465221128918.pdf]

APPENDIX

Results of Stability Testing

Detailed information of the results revealed during the two instability testing protocols is depicted in Table A1.

Table A1: Stability testing of the intact, posterior Bankart lesion, posteroinferior glenoid deficiency and posterior J-graft conditions under the two testing protocols, force-controlled and displacement-controlled, respectively.

Condition *	Posterior HH translation (mm)	Peak translational force (N)	P (posterior HH translation)§	P (peak translational force)§
Intact, 0° retroversion	1.8 ±0.8	25 ±4	n.a.	n.a.
Intact, 10° retroversion	4.0 ±3.2	21 ±6	0.124	0.026
Intact, 20° retroversion	5.4 ±2.6	19 ±6	0.016	0.011
Posterior Bankart, 0° retroversion	7.1 ±3.2	18 ±3	0.004	0.014
Posterior Bankart, 10° retroversion	7.8 ±1.9	16 ±3	<0.001	0.005
Posterior Bankart, 20° retroversion	8.2 ±1.8	13 ±2	<0.001	<0.001
20% glenoid deficiency, 0° retroversion	9.1 ±1.8	13 ±3	<0.001	<0.001
20% glenoid deficiency, 10° retroversion	9.7 ±1.1	12 ±3	<0.001	<0.001
20% glenoid deficiency, 20° retroversion	10.6 ±0.5	10 ±3	<0.001	<0.001
Posterior J-graft, 0° retroversion	2.2 ±0.7	24 ±4	0.339	0.597

HH, humeral head

* Data are presented as mean ± standard deviation

§ Each comparison is made with the intact condition at 0° retroversion and all P values are based on repeated measures analysis of variance (ANOVA; parametric data) and Friedman's ANOVA (non-parametric data) with Bonferroni adjustments.
